# Supplementary material for: Activity in perirhinal and entorhinal cortex predicts perceived visual similarities among category exemplars with highest precision
Source: eLife. 2022 Mar 21;11:e66884. doi: 10.7554/eLife.66884 (PMC9020819; doi:10.7554/eLife.66884)
Supplement: Supplementary file 1. — Proportion of correct responses for each trial type are indicated in green. [file elife-66884-supp1.docx]

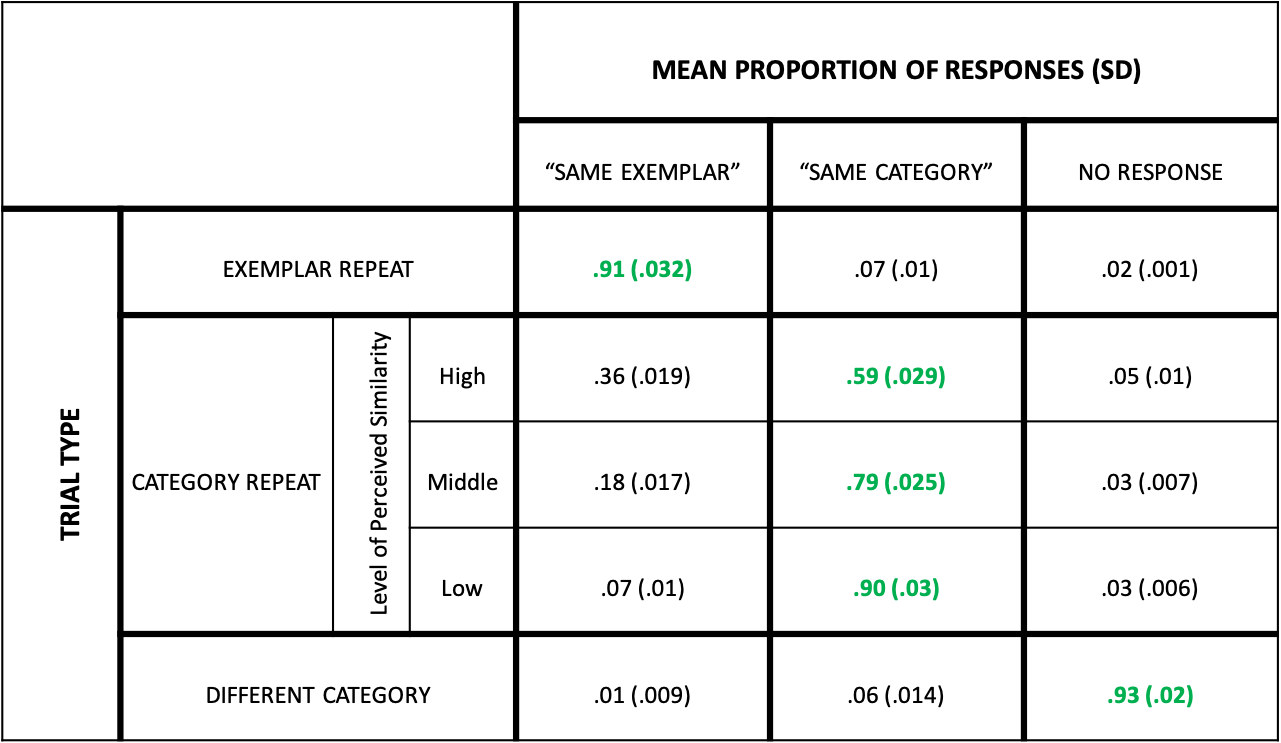


**Supplementary Table 1**. Behavioural performance on Category-Exemplar 1-Back Task. Each object image belonged to one of three trial types: exemplar repeat (Same Exemplar), category repeat (Same Category, different exemplar), no repeat (Different Category, different exemplar). Proportion of correct responses for each trial type are indicated in green. Same Category trial types are further divided according to level of perceived visual similarity, measured using participants ratings on the iMDS object sorting task.

Behavioural performance reveals sensitivity to perceived similarity in terms of accuracy of responses.
