# Supplementary material for: Activity in perirhinal and entorhinal cortex predicts perceived visual similarities among category exemplars with highest precision
Source: eLife. 2022 Mar 21;11:e66884. doi: 10.7554/eLife.66884 (PMC9020819; doi:10.7554/eLife.66884)
Supplement: Supplementary file 2. [file elife-66884-supp2.docx]

|  | **Beta (own)** | | **Beta (average)** | | **Beta (own) – Beta (average)** | |
| --- | --- | --- | --- | --- | --- | --- |
|  | **T-value** | **P-value** | **T-value** | **P-value** | **T-value** | **P-value** |
| **EVC** | 4.56 | **0.007*** | 5.12 | **0.0057*** | 0.65 | 0.600 |
| **LOC** | 4.99 | **0.0105*** | 7.43 | **0.00098*** | 1.55 | 0.10 |
| **PrC** | 7.01 | **0.0024*** | 3.06 | **0.0686** | 4.83 | **0.008*** |
| **alErC** | 6.50 | **0.0202*** | 3.01 | **0.0699** | 4.59 | **0.007*** |
| **pmErC** | 0.98 | 0.59 | 1.53 | 0.23 | 1.09 | 0.39 |
| **PhC** | 1.13 | 0.42 | 1.13 | 0.37 | 1.70 | 0.25 |
| **TP** | 1.74 | 0.12 | 1.74 | 0.22 | 0.95 | 0.50 |

**Supplementary Table 2.** Multiple linear regression: Brain RDM ~ (own RDM + average RDM). To determine the contributions of shared perceived similarity versus observer-specific perceived similarity to activity patterns in our regions of interest (ROIs), we conducted multiple regression analyses for the relationships depicted in Figure 3D using average similarity ratings and observer’s own ratings as predictors. Beta-values were tested against 0 in ROI using paired t-tests with Bonferroni-correction. Beta(own) was significantly higher than 0 in EVC, LOC, PRC, and alErC. Beta(average) was significant in EVC and LOC, but not PRC, alErC, nor in the other ROIs. Critically, the difference between Beta(own) and Beta(average) was significant only in PrC and alErC. The latter pattern of results converges with the results obtained based on calculation of the i-index as shown in Figure 7B in the main text.
