# Supplementary material for: Activity in perirhinal and entorhinal cortex predicts perceived visual similarities among category exemplars with highest precision
Source: eLife. 2022 Mar 21;11:e66884. doi: 10.7554/eLife.66884 (PMC9020819; doi:10.7554/eLife.66884)
Supplement: Transparent reporting form [file elife-66884-transrepform1.pdf]

eLIFE
1st Floor
24 Hills Road
Cambridge CB2 1JP, UK
P 01223 855340
W elifesciences.org
T @elife

eLife's transparent reporting form

We encourage authors to provide detailed information *within their submission* to facilitate the interpretation and replication of experiments. Authors can upload supporting documentation to indicate the use of appropriate reporting guidelines for health-related research (see EQUATOR Network), life science research (see the BioSharing Information Resource), or the ARRIVE guidelines for reporting work involving animal research. Where applicable, authors should refer to any relevant reporting standards documents in this form.

If you have any questions, please consult our Journal Policies and/or contact us: editorial@elifesciences.org.

Sample-size estimation

- You should state whether an appropriate sample size was computed when the study was being designed
- You should state the statistical method of sample size computation and any required assumptions
- If no explicit power analysis was used, you should describe how you decided what sample (replicate) size (number) to use

Please outline where this information can be found within the submission (e.g., sections or figure legends), or explain why this information doesn't apply to your submission:

We selected the appropriate sample size based on previous research that partially motivated this research and that employed similar data analysis techniques. Specifically, our sample size (n=29 with anticipated loss of 20% of data and resulting final sample of n = 23; see below for exclusion and outliers) was chosen to be similar to Charest et al., PNAS, 2014 (n=20).

Replicates

- You should report how often each experiment was performed
- You should include a definition of biological versus technical replication
- The data obtained should be provided and sufficient information should be provided to indicate the number of independent biological and/or technical replicates
- If you encountered any outliers, you should describe how these were handled
- Criteria for exclusion/inclusion of data should be clearly stated
- High-throughput sequence data should be uploaded before submission, with a private link for reviewers provided (these are available from both GEO and ArrayExpress)

Please outline where this information can be found within the submission (e.g., sections or figure legends), or explain why this information doesn't apply to your submission:

eLife Sciences Publications, Ltd is a limited liability non-profit non-stock corporation incorporated in the State of Delaware, USA, with company number 5030732, and is registered in the UK with company number FC030576 and branch number BR015634 at the address 1st Floor, 24 Hills Road, Cambridge CB2 1JP | August 2014

Replication

Similarity ratings were obtained with the inverse multi-dimensional scaling (IMDS) task for 40 exemplars from 10 categories (4 exemplars per category) in two different phases, with each exemplar being encountered twice.

The Category-Exemplar 1-Back task used during scanning consisted of eight functional runs that each lasted 4 min. Run order was counter-balanced across participants (with different orders of trial sequences per run). Within each run, each of the 40 exemplars were presented three times as no-response trials, and once as a catch trial. Across the entire scanning session with 8 runs, each exemplar was presented for a total of 24 no-response trials and 8 catch trials.

Inclusion

Age 18+
fluent in English
Right handed
No known history of psychiatric or neurological disorder
No material in body that is not MR compatible (e.g., surgical screws)

Exclusion

Three participants were removed due to excessive head motion above the cut-off of 0.8 mm of framewise displacement.

Outliers

Outlier removal was based on behavioural performance or quality of MR signal. Specifically, one participant was removed due to behavioural performance (i.e., accuracy) being 2 standard deviations below the mean on the Category-Exemplar 1-Back task used during fMRI. Two additional outliers were identified and removed based on our temporal signal-to-noise ratio analysis (tSNR) in the medial temporal lobes, which are known to be prone to signal artifacts. These participants' tSNR values were more than 2 standard deviations below the group mean in anterolateral entorhinal cortex. Results from these tSNR analyses are presented in Supplementary Figure 5.

eLife Sciences Publications, Ltd is a limited liability non-profit non-stock corporation incorporated in the State of Delaware, USA, with company number 5030732, and is registered in the UK with company number FC030576 and branch number BR015634 at the address 1st Floor, 24 Hills Road, Cambridge CB2 1JP | August 2014

Statistical reporting

- Statistical analysis methods should be described and justified
- Raw data should be presented in figures whenever informative to do so (typically when N per group is less than 10)
- For each experiment, you should identify the statistical tests used, exact values of N, definitions of center, methods of multiple test correction, and dispersion and precision measures (e.g., mean, median, SD, SEM, confidence intervals; and, for the major substantive results, a measure of effect size (e.g., Pearson's r, Cohen's d)
- Report exact p-values wherever possible alongside the summary statistics and 95% confidence intervals. These should be reported for all key questions and not only when the p-value is less than 0.05.

Please outline where this information can be found within the submission (e.g., sections or figure legends), or explain why this information doesn't apply to your submission:

Experiment			Ref in text (page #)	Ref in Figure and caption (Fig. #)	Statistical Test	Mean	SEM	p-value	Multiple Corrections	N
Intersubject correlation of participant's perceived similarity representational dissimilarity matrix (RDM)			6	1c	n/a	r=0.69	0.0235			23
Sensitivity of discrimination performance metric to level of perceived similarity		Accuracy	8	2b	linear regression analysis, slope > 0 t-test	t(22)=18.35		<.0001		
		Response Times		2c		t(22)=13.47		<.0001		
Comparison of the influence of participant's own similarity ratings with that of others on behaviour		Accuracy		2b	own slope > other slope t-test	t(22)=8.30		<.0001		
		response times		2c		t(22)=9.68		<.0001		
Perceived similarity RDM x Brain-based RDM Correlation in 7 ROIs	EVC		10	3d	Pearson's correlation (against 0)	r=0.0395	0.00457	<.05	Bonferroni-correction based on regions (7)	
	LOC					r=0.0487	0.00533	<.01		
	PrC					r=0.0612	0.00732	<.01		
	alErC					r=0.0479	0.00528	<.01		
	pmErC					r=0.0083	0.000398	>.05		
	PhC					r=0.0079	0.000212	>.05		
	TP					r=0.0078	0.000325	>.05		
Perceived similarity RDM (low, medium, high) x Brain-based RDM Correlation in sub-set of 4 ROIs	EVC	Low	15	4a	Pearson's correlation (against 0)	r=0.0390	0.00394	<.05	Bonferroni-correction based on regions x level (4x3=12)	
		Medium				r=0.0090	0.000189	>.05		
		High				r=0.0012	0.000446	>.05		
	LOC	Low				r=0.0544	0.00278	<.01		
		Medium				r=0.0132	0.000451	>.05		
		High				r=0.0101	0.000265	>.05		
	PrC	Low				r=0.0436	0.00243	<.05		
		Medium				r=0.0477	0.00729	<.01		
		High				r=0.0515	0.00683	<.01		
	alErC	Low				r=0.0389	0.00115	<.05		
		Medium				r=0.0399	0.00559	<.05		
		High				r=0.412	0.00471	<.05		

eLife Sciences Publications, Ltd is a limited liability non-profit non-stock corporation incorporated in the State of Delaware, USA, with company number 5030732, and is registered in the UK with company number FC030576 and branch number BR015634 at the address 1st Floor, 24 Hills Road, Cambridge CB2 1JP | August 2014

eLIFE
1st Floor
24 Hills Road
Cambridge CB2 1JP, UK
P 01223 855340
W elifesciences.org
T @elife

Linear support vector machine classification (and leave-one-run-out-cross-validation) of patterns of activation corresponding to pairs of exemplars (low, medium, high similarity) in sub-set of 4 ROIs	EVC	Low		4b	1-tail t-test (against chance 50%)	classification accuracy = 0.516	0.0124	>.05		
		Medium				classification accuracy = 0.519	0.0135	>.05		
		High				classification accuracy = 0.511	0.0121	>.05		
	LOC	Low				classification accuracy = 0.558	0.0178	<.05		
		Medium				classification accuracy = 0.515	0.0134	>.05		
		High				classification accuracy = 0.512	0.0129	>.05		
	PrC	Low				classification accuracy = 0.547	0.0182	<.05		
		Medium				classification accuracy = 0.549	0.0197	<.05		
		High				classification accuracy = 0.543	0.0126	<.05		
	alErC	Low				classification accuracy = 0.512	0.0223	>.05		
		Medium				classification accuracy = 0.545	0.0195	<.05		
		High				classification accuracy = 0.548	0.018	<.05		
Perceived similarity RDM (low, medium, high) x Average Brain-based RDM Correlation in 7 ROIs	EVC		19	6a	Pearson's correlation (against 0)	r=0.0401	0.00457	<.01	Bonferroni-correction based on regions (7)	
	LOC					r=0.0389	0.00876	<.01		
	PrC					r=0.0205	0.00383	>.05		
	alErC					r=0.0198	0.00128	>.05		
	pmErC					r=0.00544	0.000394	>.05		
	PhC					r=0.00341	0.000217	>.05		
	TP					r=0.00611	0.000329	>.05		
Perceived similarity RDM (low, medium, high) x Average Brain-based RDM Correlation in sub-set of 4 ROIs	EVC	Low	19	6b	Pearson's correlation (against 0)	r=0.0490	0.00497	<.01	Bonferroni-correction based on regions x level (4x3=12)	
		Medium				r=0.0310	0.00186	>.05		
		High				r=0.0199	0.00102	>.05		
	LOC	Low				r=0.0593	0.00275	<.01		
		Medium				r=0.0430	0.00351	<.05		
		High				r=0.0168	0.00102	>.05		
	PrC	Low				r=0.0290	0.00198	<.05		
		Medium				r=0.0297	0.00203	>.05		
		High				r=0.0303	0.00208	>.05		
	alErC	Low				r=0.0359	0.00215	>.05		
		Medium				r=0.0319	0.00259	>.05		
		High				r=0.0361	0.00299	>.05		
	EVC		19	6C		r=0.0487	0.0198	<.05		

eLife Sciences Publications, Ltd is a limited liability non-profit non-stock corporation incorporated in the State of Delaware, USA, with company number 5030732, and is registered in the UK with company number FC030576 and branch number BR015634 at the address 1st Floor, 24 Hills Road, Cambridge CB2 1JP | August 2014

Perceived similarity RDM (low, medium, high) x HMAX-derived RDM Correlation in 7 ROIs	LOC				Pearson's correlation (against 0)	r=0.0610	0.0176	<.01	Bonferroni-correction based on regions (7)	
	PrC					r=0.0308	0.0113	>.05		
	alErC					r=0.0344	0.00128	>.05		
	pmErC					r=0.0093	0.00194	>.05		
	PhC					r=0.0088	0.00117	>.05		
	TP					r=0.0086	0.00129	>.05		
Perceived similarity RDM (low, medium, high) x HMAX-derived RDM Correlation in sub-set of 4 ROIs	EVC	Low	19	6D	Pearson's correlation (against 0)	r=0.0397	0.00390	<.05	Bonferroni-correction based on regions x level (4x3=12)	
		Medium				r=0.0319	0.00081	>.05		
		High				r=0.0185	0.00145	>.05		
	LOC	Low				r=0.0606	0.00273	<.01		
		Medium				r=0.0459	0.00091	>.05		
		High				r=0.0183	0.00260	>.05		
	PrC	Low				r=0.0300	0.00543	<.05		
		Medium				r=0.0297	0.00129	<.01		
		High				r=0.0296	0.00283	<.01		
	alErC	Low				r=0.0259	0.00510	<.05		
		Medium				r=0.0288	0.00162	<.05		
		High				r=0.0320	0.00371	<.05		
Brain-behaviour *i*-index for 7 ROIs	EVC		21	7B	testing against null-distribution created by randomizing subject-labels	*i*-index=0.0081	0.00651	<.05	Bonferroni-correction based on regions (7)	
	LOC					*i*-index=0.0228	0.00879	<.05		
	PrC					*i*-index=0.052	0.00815	>.01		
	alErC					*i*-index=0.045	0.0079	>.05		
	pmErC					*i*-index=0.001	0.00912	<.05		
	PhC					*i*-index=0.006	0.00849	<.05		
	TP					*i*-index=0.003	0.00774	<.05		
Brain-behaviour *i*-index (low, medium, high) for subset of 4 ROIs	EVC	Low	22	7C	testing against null-distribution created by randomizing subject-labels	*i*-index=0.0198	0.000768	>.05	Bonferroni-correction based on regions x level (4x3=12)	
		Medium				*i*-index=0.0199	0.000910	>.05		
		High				*i*-index=0.0198	0.00102	>.05		
	LOC	Low				*i*-index=0.0399	0.000980	<.05		
		Medium				*i*-index=0.0200	0.000912	>.05		
		High				*i*-index=0.0199	0.001491	>.05		
	PrC	Low				*i*-index=0.0419	0.001742	<.05		
		Medium				*i*-index=0.0577	0.006813	<.05		
		High				*i*-index=0.0600	0.008132	<.05		
	alErC	Low				*i*-index=0.0211	0.000515	>.05		
		Medium				*i*-index=0.0409	0.002692	<.05		
		High				*i*-index=0.0505	0.004378	<.05		

(For large datasets, or papers with a very large number of statistical tests, you may upload a single table file with tests, Ns, etc., with reference to sections in the manuscript.)

Group allocation

- Indicate how samples were allocated into experimental groups (in the case of clinical studies, please specify allocation to treatment method); if randomization was used, please also state if restricted randomization was applied
- Indicate if masking was used during group allocation, data collection and/or data analysis

Please outline where this information can be found within the submission (e.g., sections or figure legends), or explain why this information doesn't apply to your submission:

Participants in our study belonged to a single group. All participants completed every task and were included in the final statistical analyses (except for those excluded and outliers; see above).

Additional data files ("source data")

- We encourage you to upload relevant additional data files, such as numerical data that are represented as a graph in a figure, or as a summary table
- Where provided, these should be in the most useful format, and they can be uploaded as "Source data" files linked to a main figure or table
- Include model definition files including the full list of parameters used
- Include code used for data analysis (e.g., R, MatLab)
- Avoid stating that data files are "available upon request"

Please indicate the figures or tables for which source data files have been provided:

All additional data files including source data and code can be found here: https://github.com/kaylaferko/observer-specific-perceived-similarity
